# London Cow-Houses

**Published:** 1858-04

**Authors:** S. R. Bristowe, Edw. Ballard

**Affiliations:** Secretary.; Chairman.


### LONDON COW-HOUSES.

A COMMITTEE appointed by the Metropolitan Association of Medical Officers of Health, to report on the London cow-houses, have produced a short but useful report, from which a few of the more interesting particulars may be copied with advantage.

The number of establishments comprised in the returns collected by the committee amounted to 846, and the number of cows to 11,818. The dimensions of the sheds for the cows varied. To obtain accuracy, the committee calculated the cubic space allowed to each cow. The space allotted to each animal increases gradually from 200 feet to between 300 and 400. The paving was good in 554 cases, indifferent in 133 cases, and bad in 199. The drainage was good in 503 cases, indifferent in 103, and bad in 156. The ventilation was good in 528 instances, indifferent in 152, and bad in 122. The houses were free from bad smell in 463 cases, indifferent in 137, and offensive in 108.

In 71 cases there were no yards, or other open spaces, connected with the establishment; in 390 the yards are said to have been in good condition; in 137 they are stated indifferent; and in 172 bad.

The health of the cows is reported to have been good in 637 cases, indifferent in 83, and bad in 7. It was good in 87 per cent. of the entire number of establishments reported on. It was good in 86 per cent. of those with from 1 to 5 cows each; in 89 per cent. of those with from 6 to 10; in 84 per cent. of those with from 11 to 20; and in 87 of those with from 21 upwards. In some of the cases in which the health is reported to have been indifferent, it is stated merely that the cows looked out of condition; and in several instances in which the health is reported to have been good, it is mentioned (occasionally with details) that the health had been bad or indifferent at some antecedent period, varying from a few weeks to a few years back.

Under the head of health of cows, the information received is not trustworthy; for the health would appear, from the return, to be exceedingly good, and to be equally favourable in establishments of all sizes; whereas there are strong grounds for believing the direct contrary to be the fact: and, indeed, in one particular, no doubt, the returns are unfair to the larger establishments; for when the health is stated, in the answers, to be bad or indifferent, this statement is generally founded on the fact of *present* illness or indisposition in one or more of the cows; and thus, on this account, establishments with many cows necessarily incurred a greater risk than others of being reckoned unhealthy. The truth is, that cow-keepers generally have, for obvious reasons, been very reserved on this subject, though, in some few instances, admissions have been made which are somewhat remarkable. Thus, one proprietor with 7 cows allows that he has lost 10, within the last seven months, from lung disease; another, with 4 cows, admits that 10 have died of the same complaint within the last three months; and one with 8 states that he has lost the enormous number of 230 in the course of twelve years.

There are several other facts, however, with regard to the health of cows, which, though not appearing in any numerical strength from the returns, are known, from other sources of information, to be true, and are of considerable importance as confirming statements made in the preceding paragraph. In the first place, it is well known that when cows are attacked with any serious acute disease, it is more advantageous to their owners to dispose of them at once to the butchers, than to keep them through an unprofitable illness, with the risk of death at the end; and, moreover, that there are certain persons, whose business it is to visit cow-houses periodically and frequently for the purpose of buying such animals as are out of health. Secondly, it is a significant fact that the rate of insurance imposed on cows is 7½*d.* in the pound for those in the country, whilst it varies between 1*s.* 6*d.* and 2*s.* in the pound for those in London, a difference said to be "dependent on the mode of lodgment and general management."

The Committee, in conclusion, offer the following rules for the regulations for cow-houses.

1. Every cow-house shall be paved with flag-paving, or other non-absorbent material, set and bedded in cement, with a proper inclination to the foot of the stalls, so as to drain into a channel leading by a fall of not less than 1½ inches in 10 feet to a trapped gulley.

2. Every cow-house shall be provided with a proper trapped drain, to convey fluid matter alone into the sewers.

3. Every cow-house shall be furnished with an adequate supply of water, and be washed thoroughly at least once a day.

4. All solid manure and refuse shall be carefully swept up twice a day, be kept under cover, and be carted away every morning by seven o'clock from Lady-day to Michaelmas, and by eight o'clock from Michaelmas to Lady-day.

5. Every cow-house shall be kept in proper condition, and the walls be limewashed at least four times a year, within fourteen days after the quarter.

6. Every cow-house shall have a space of at least 8 feet by 4 feet for each cow, (when the cows are kept in separate stalls), or of 8 feet by 7 feet for every two cows, (when the stalls are constructed to hold pairs), with a cubic capacity, in either case, of at least 1000 feet to each cow; shall be properly lighted and ventilated; and when the state of the neighbourhood requires it, shall be provided with tight roofs and ventilating shafts, so as to convey the noxious exhalations above the level of the adjacent houses.

7. Every yard, in which a cow-house is situated, shall be well paved with stone or other impervious material (the joints of the paving to be run with grout), with such a slope towards the channels and trapped gulley as to permit the rapid escape of all fluids into the sewer; and shall be washed at least once a day.

8. The grain bins and receptacles for wash shall be kept properly cleansed and under cover.

9. No underground cellar, and no part of a dwellinghouse, shall be used as cowsheds.

*Signed*, S. R. BRISTOWE, *Secretary.*

EDW. BALLARD, *Chairman.*

---

